# Supplementary material for: Integrated Metabolomics and Transcriptomics Analyses Identify Key Amino Acid Metabolic Mechanisms in Lacticaseibacillus paracasei SMN-LBK
Source: Foods. 2025 Feb 21;14(5):730. doi: 10.3390/foods14050730 (PMC11899468; doi:10.3390/foods14050730)
Supplement: Supplementary file 1 [file foods-14-00730-s001.zip › foods-3452482-supplementary.pdf]

A

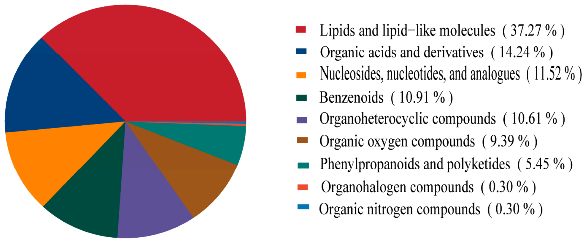

B

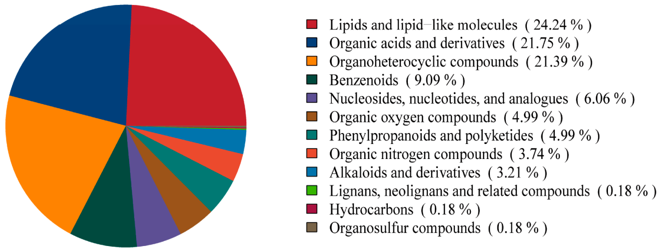

Figure S1. Metabolite classification pie charts. (A) Pie chart in negative ion mode. (B) Pie chart in positive ion mode.

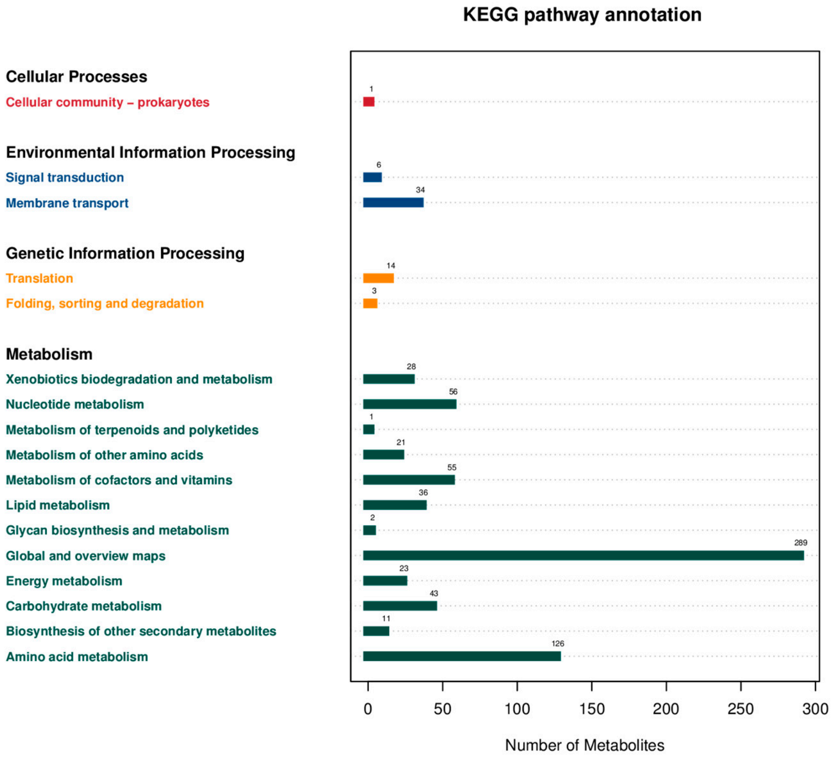

Figure S2. KEGG pathway annotation diagram.

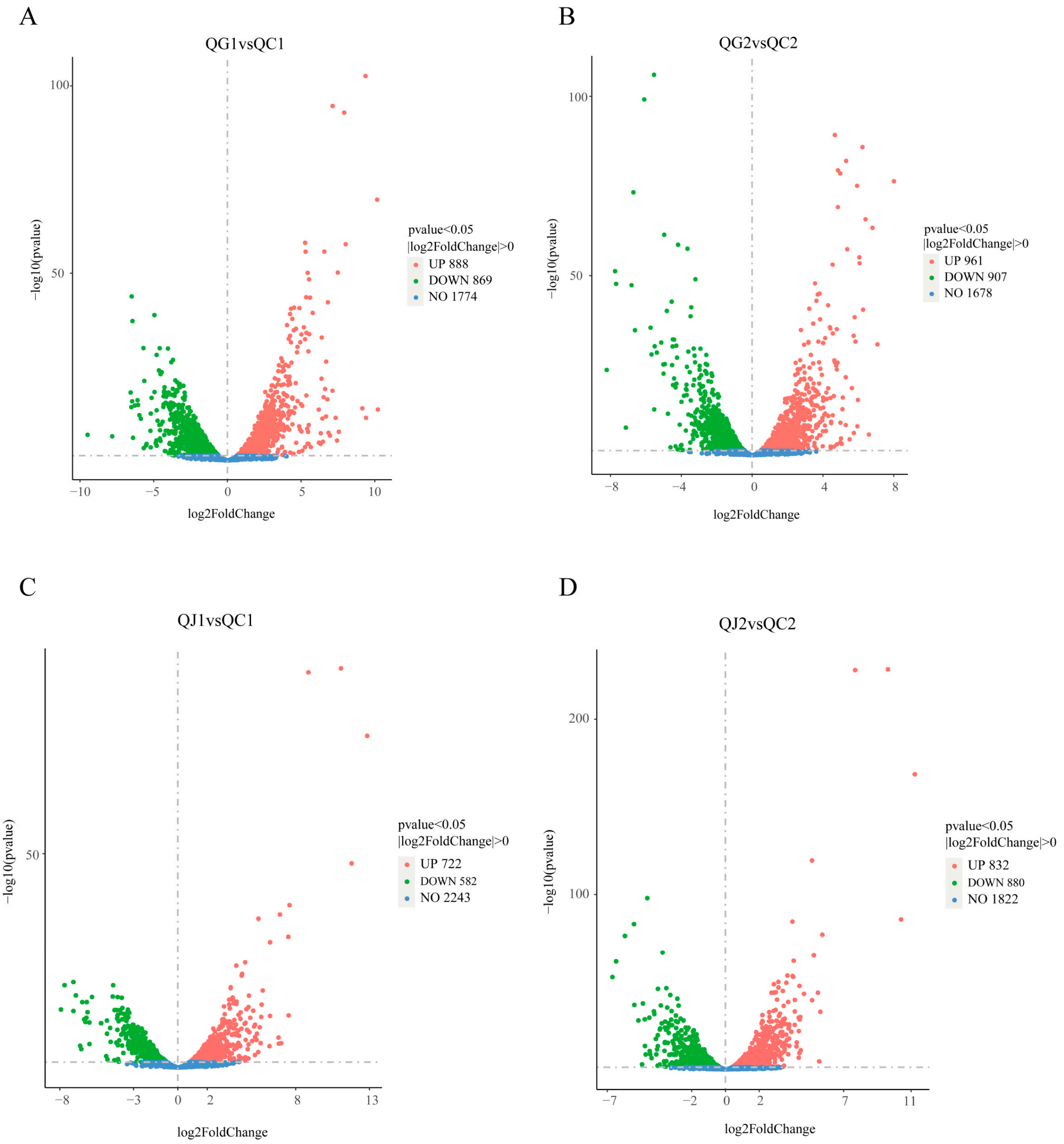

Figure S3. Comparison of differential genes between different groups. (A-D) Volcano plots depicting the differential genes across the four comparative pairs. QG, QJ, and QC denote the glutamate-deficient group, arginine-deficient group, and CDM group, respectively. The number 1 indicates a logarithmic growth period (10h) and 2 indicates a stable growth period (18h). The red point represents the difference is up-regulated, the green point represents the difference is down-regulated, and the blue point represents the metabolite that does not meet the different screening conditions.

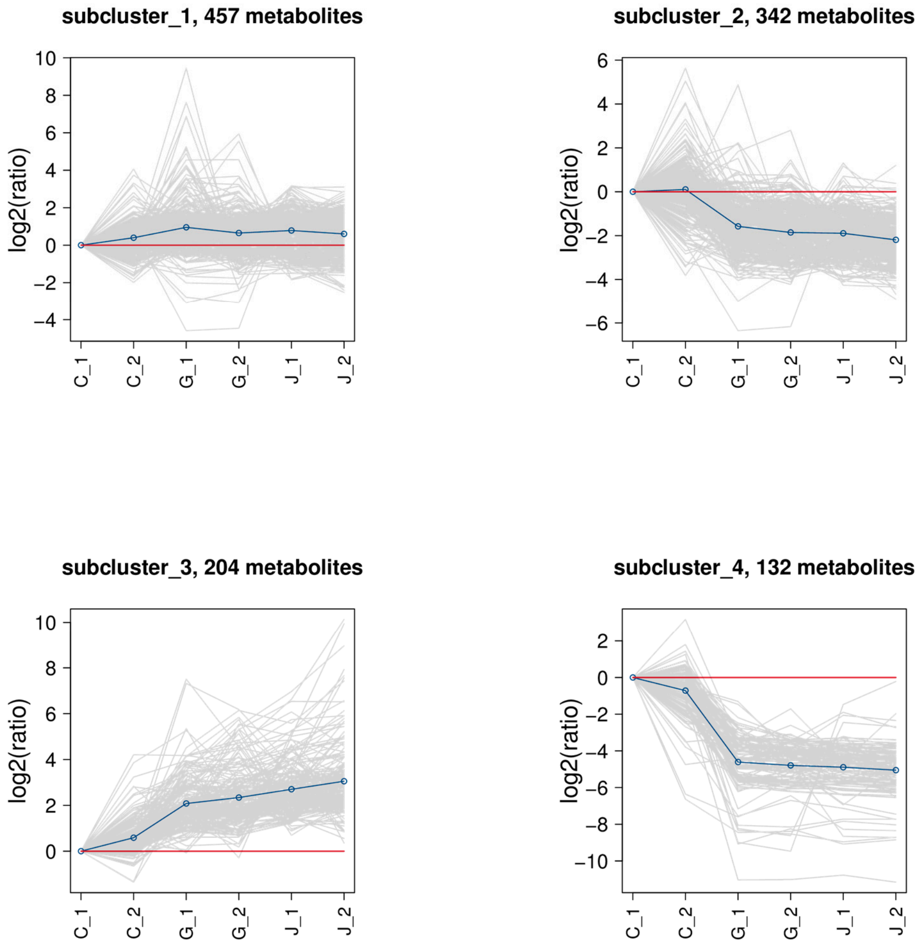

Figure S4. K-Means analysis of differential metabolites. experimental groups G and J consisted of SMN-LBK cultured in glutamate-deficient and arginine-deficient CDM medium, respectively. Control groups C consisted of SMN-LBK cultured in CDM medium. The number 1 indicates a logarithmic growth period (10h) and 2 indicates a stable growth period (18h).

Table S1. Number of differential genes enriched for selected metabolic pathways

QG1 VS QC1		QG2 VS QC2	
Description	GeneRatio	Description	GeneRatio
Pyrimidine metabolism	23/397	Pyruvate metabolism	20/411
Alanine, aspartate and glutamate metabolism	13/397	Alanine, aspartate and glutamate metabolism	12/411
Glycolysis / Gluconeogenesis	23/397	Glycolysis / Gluconeogenesis	23/411
Pyruvate metabolism	14/397	Pentose phosphate pathway	19/411
Purine metabolism	15/397	Pyrimidine metabolism	16/411
Pentose phosphate pathway	13/397	Purine metabolism	16/411
QJ1 VS QC1		QJ2 VS QC2	
Description	GeneRatio	Description	GeneRatio
Alanine, aspartate and glutamate metabolism	12/277	Pentose phosphate pathway	22/363
Pyrimidine metabolism	16/277	Purine metabolism	18/363
Purine metabolism	12/277	Glycolysis / Gluconeogenesis	21/363
Pentose phosphate pathway	9/277	Alanine, aspartate and glutamate metabolism	10/363
Pyruvate metabolism	4/277	Pyruvate metabolism	11/363
Glycolysis / Gluconeogenesis	7/277	Pyrimidine metabolism	10/363

Note: GeneRatio represents the ratio of the number of differential genes annotated to the KEGG pathway to the total number of differential genes. QG, QJ, and QC denote the glutamate-deficient group, arginine-deficient group, and CDM group, respectively. The number 1 after the letter indicates a logarithmic growth period (10h) and 2 indicates a stable growth period (18h).
